# A multicenter, retrospective study of cardiac disease in Borzoi dogs

**DOI:** 10.3389/fvets.2023.1102494

**Published:** 2023-01-26

**Authors:** K. Tess Sykes, Sonya Wesselowski, Ashley B. Saunders, Sonja S. Tjostheim, Brianna M. Potter, Anna R. M. Gelzer, Natalie Katz, Jessica L. Ward, Emily T. Karlin, Lauren E. Markovic, Aliya N. Magee, Jonathan A. Abbott, Saki Kadotani, Giulio Menciotti

**Affiliations:** ^1^Department of Small Animal Clinical Sciences, College of Veterinary Medicine, Texas A&M University, College Station, TX, United States; ^2^Department of Medical Sciences, School of Veterinary Medicine, University of Wisconsin, Madison, WI, United States; ^3^Department of Clinical Sciences, College of Veterinary Medicine and Biomedical Sciences, Colorado State University, Fort Collins, CO, United States; ^4^Department of Clinical Studies and Advanced Medicine, School of Veterinary Medicine, University of Pennsylvania, Philadelphia, PA, United States; ^5^College of Veterinary Medicine, Cornell University, Ithaca, NY, United States; ^6^Department of Veterinary Clinical Sciences, College of Veterinary Medicine, Iowa State University, Ames, IA, United States; ^7^Department of Clinical Sciences, Cummings School of Veterinary Medicine, Tufts University, North Grafton, MA, United States; ^8^Department of Small Animal Medicine and Surgery, College of Veterinary Medicine, University of Georgia, Athens, GA, United States; ^9^Department of Veterinary Clinical Sciences, School of Veterinary Medicine, Louisiana State University, Baton Rouge, LA, United States; ^10^Department of Small Animal Clinical Sciences, College of Veterinary Medicine, University of Tennessee, Knoxville, Knoxville, TN, United States; ^11^Department of Veterinary Clinical Medicine, College of Veterinary Medicine, University of Illinois Urbana-Champaign, Urbana, IL, United States; ^12^Department of Small Animal Clinical Sciences, Virginia-Maryland College of Veterinary Medicine, Blacksburg, VA, United States

**Keywords:** sighthound, dilated cardiomyopathy, ventricular arrhythmias, sudden death, tricuspid regurgitation, mitral regurgitation

## Abstract

Borzoi are large, relatively uncommon sighthounds anecdotally reported to suffer from sudden death. This multicenter retrospective cohort study aimed to describe the sample of Borzoi presenting to veterinary cardiologists for evaluation, with records searched from 14 centers across a study period of up to 20 years. The study sample was comprised of 152 client-owned Borzoi, with dogs most commonly presenting for pre-breed screening in 87/152 (52%), followed by evaluation of an arrhythmia in 28/152 (18%). Of the 131/152 (86%) dogs that had an echocardiogram performed, 85/131 (65%) were structurally normal, with 40/85 (47%) structurally normal dogs having trace or mild atrioventricular valve regurgitation. Tricuspid valve dysplasia was the most commonly diagnosed congenital cardiac disease (*n* = 6). Myxomatous mitral valve disease (*n* = 12) and dilated cardiomyopathy (*n* = 13) were diagnosed at similar frequencies, though 92% of valve disease cases were mild. Only 48/152 (32%) Borzoi had a diagnostic electrocardiogram (ECG) and/or a Holter monitor for arrhythmia screening. Despite this, ventricular arrhythmias were identified during the entirety of the available cardiac evaluation including diagnostic ECG, contemporaneous ECG monitoring during the echocardiogram, and/or Holter monitor in 25/131 (19%) dogs in which an echocardiographic diagnosis was available. Of these 25 Borzoi, 76% had minimal or no structural cardiac disease identified, and five had a family history of sudden death. A sudden death outcome was reported in 3/55 (5%) Borzoi with long-term outcome data available. In conclusion, Borzoi commonly have trace or mild atrioventricular valve insufficiencies, and may develop ventricular arrhythmias and dilated cardiomyopathy.

## 1. Introduction

Borzoi are large, relatively uncommon sighthounds that are bred to be both fast and powerful and frequently compete in a variety of athletic events. In 2020, the American Kennel Club ranked Borzoi as the 103rd most registered breed, out of 195. Given their overall rarity, broad clinical experience with Borzoi for any individual veterinarian is unlikely. Amongst Borzoi breeders and owners, however, sudden, unexplained death is a recognized concern, with an active push for affected dogs to be examined postmortem (1, 2). Unfortunately, definitive data as to the cause of sudden death in the breed is currently lacking. In a study analyzing cause of death in dogs at 27 participating veterinary teaching hospitals over a 20-year period, 104 Borzoi were identified (3). Of the 104 Borzoi that died, the leading causes of death grouped by organ system were respiratory and musculoskeletal (both at 16.3%), while cardiovascular deaths made up only 6.7% (3). Unfortunately, the majority of sudden cardiac deaths are unlikely to be accounted for in a hospital setting.

Prior publications have demonstrated that sighthounds have echocardiographic measurements that differ from non-sighthound breeds (4–11), and that they frequently have physiologic heart murmurs (12–14). A high incidence of trivial atrioventricular valve insufficiencies and myxomatous mitral valve disease (MMVD) has been reported in Whippets (13), while a predisposition for developing familial dilated cardiomyopathy (DCM) is well-described in Irish Wolfhounds (15–18) and Scottish Deerhounds (19), both large sighthound breeds. To date, there are no data available to define the overall prevalence of cardiac disease in the Borzoi breed or to identify the types of cardiac disease that they may be predisposed to, with the exception of a single Borzoi diagnosed with tricuspid valve dysplasia (TVD) in one retrospective study (20) and a single Borzoi diagnosed with a patent ductus arteriosus (PDA) in another retrospective study (21). Additionally, no breed-specific echocardiography reference intervals are currently available for Borzoi.

The objective of the present study was to describe the sample of Borzoi that presented to veterinary cardiologists for evaluation over a 20-year period. We hypothesized that both DCM and ventricular arrhythmias would be diagnosed in Borzoi dogs.

## 2. Materials and methods

Medical records from 13 Veterinary Teaching Hospitals and one multi-site private practice cardiology group were retrospectively reviewed to identify Borzoi evaluated by each hospital's cardiology service over a 20-year period (2001–2021). If available, total number of Borzoi evaluated at each hospital over the same period was also collected to determine the percentage of Borzoi that were evaluated by a cardiology service specifically, as was the total number of individual dogs of all breeds that presented to each individual site to determine the frequency with which Borzoi were evaluated for any reason. Ethical approval and client consent was not required by the lead author's institution for a retrospective study in which no research was conducted on live animals.

Information collected from each medical record included date of evaluation, date of birth, sex, body weight, body condition score, reason for cardiac evaluation, history of clinical signs, preexisting medical conditions and medications, physical examination findings, results of any cardiac diagnostic tests including echocardiograms, electrocardiograms (ECG), blood pressure (BP) measurements, thoracic radiographs, and Holter monitor recordings, cardiac medications that were started as a result of the evaluation, and if the dog was rechecked or developed cardiac disease in the future. Final diagnoses were obtained from reviewing reports for cardiac diagnostics and patient discharge summaries at the time of the initial evaluation.

Selected echocardiographic measurements were retrieved from all available echocardiogram reports. Stored echocardiographic cine loops were not reviewed or re-measured. Collected echocardiographic variables included end diastolic left ventricular diameter, end systolic left ventricular diameter, fractional shortening (FS), ejection fraction by Simpson's method of disks (EF), left atrium to aortic root ratio, the presence and quantification of valvular insufficiencies, provided description(s) of valve anatomy, tricuspid regurgitation velocity, transaortic velocity, transpulmonic velocity, the presence and quantity of any identified effusions and the cardiac rhythm documented during the contemporaneous ECG associated with the echocardiogram. For dogs in which echocardiographic measurement data were available, these data were reviewed, and dogs were reclassified using a single set of criteria to categorize dogs as having normal left ventricular dimensions and systolic function vs. equivocal, occult or overt DCM. Left ventricular dimensions at end-diastole and end-systole were normalized to body weight using previously published allometric scaling exponents (22). A diagnosis of occult DCM required that normalized left ventricular dimensions at end-systole exceed the upper 97.5 percentile prediction interval of 1.26, with or without the normalized left ventricular dimension at end-diastole exceeding the equivalent cut-off of 1.85. Additionally, a FS < 20–25% and an EF < 40% were considered supportive findings, as defined in a recent review on screening for DCM in dogs (23), with one or both of these findings confirmed in all dogs categorized as occult DCM for this study. Dogs with overt DCM met the same echocardiographic criteria as those outlined for occult DCM but also had evidence of active or medically managed congestive heart failure or had evidence of both DCM and congestive heart failure on necropsy based on the discretion of the attending pathologist. Dogs with left ventricular dilation at end-systole and end-diastole but a FS ≥ 25% and an ejection fraction ≥ 40% in the absence of moderate to severe mitral regurgitation (MR) were categorized as equivocal DCM, as were dogs with normalized left ventricular end-diastolic and end-systolic dimensions within the 2.5–97.5 percentile prediction intervals in which fractional shortening was < 20%. Dogs diagnosed with MMVD were staged based on the most recent consensus guidelines (24).

Arrhythmia diagnoses were made based on all available reports provided from the medical record. This included contemporaneous ECG recordings during the echocardiogram, diagnostic ECG, and Holter monitor reports.

Long-term follow up and cause of death was extracted from the medical record, if available. For cases in which outcome was not available, owners were contacted *via* email and/or phone to obtain additional information about the development of cardiac disease or initiation of cardiac medications in each dog, as well as their date and cause of death, if applicable. The cause of death was reported and characterized as cardiac (refractory heart failure or sudden unexplained death in the absence of other known causes) or non-cardiac, if possible.

Descriptive statistics were calculated for each of the reported variables and are reported as median (range) or number (proportion).

## 3. Results

A total of 628 Borzoi presented to the 13 academic veterinary teaching hospitals and the various locations of the multi-site private practice cardiology group within the study period, as outlined in Table 1. All Borzoi were evaluated at the primary hospital location, except for a small number of Borzoi that were evaluated offsite at cardiac breed screening events by cardiologists associated with the private practice cardiology group. The 628 identified Borzoi represented a median of 0.02% (range: 0.01–0.08%) of all canine hospital admissions from the 11 hospitals in which this could be calculated. Nine of the 14 hospitals were able to search for dogs during the entire 20-year period. The remaining five hospitals were able to search eight, 11, 15, 15, and 19 years, respectively. Of the 628 Borzoi identified within the study period, 152 dogs (24%) were evaluated by a cardiology service and comprise the study sample.

**Table 1:** Borzoi data from all participating sites over a maximum 20-year period.

	**Search period**	**Total number of Borzoi presented to hospital during search period**	**% of all canine hospital-wide admissions to hospital that were Borzoi**	**Number of Borzoi evaluated by cardiology**	**% of Borzoi admissions that were evaluated by cardiology**
Hospital 1	2001–2021	24	0.06%	9	38%
Hospital 2	2001–2021	30	0.04%	2	7%
Hospital 3	2001–2021	49	0.03%	7	14%
Hospital 4	2002–2021	58	0.08%	23	40%
Hospital 5	2001–2021	34	0.01%	8	24%
Hospital 6	2001–2021	183	0.03%	15	5%
Hospital 7	2013–2021	24	0.02%	10	42%
Hospital 8^*^	2006–2021	39	N/A	39	100%
Hospital 9	2001–2021	25	0.02%	7	28%
Hospital 10	2001–2021	35	N/A	2	6%
Hospital 11	2001–2021	69	0.01%	10	14%
Hospital 12	2001–2021	12	0.01%	2	17%
Hospital 13	2010–2021	29	0.03%	13	41%
Hospital 14	2006–2021	17	0.01%	5	29%

* Hospital 8 reflects a combination of data from the multiple sites of one large private practice cardiology specialty group. N/A, not available.

### 3.1. Signalment and history

The median age at the time of evaluation was 4.4 years (range: 2 months−11.6 years), with age unable to be determined from the medical record in 14 adult dogs undergoing offsite cardiac screening. The sex distribution was relatively equal, with 69 males (54 intact) and 74 females (52 intact). Sex was not recorded in nine instances.

The majority of cases (87/152, 52%) presented for cardiac screening, including four dogs in which a family history of sudden death (*n* = 2) or DCM (*n* = 2) was recorded. The second most common reason for cardiac evaluation was work-up for a documented arrhythmia (28/152, 18%), with three of these dogs having a history of sudden death in one (*n* = 2) or two (*n* = 1) siblings noted in their history. Additional reasons for cardiac evaluation included evaluation of a heart murmur (21/152, 14%), history of respiratory signs including cough, dyspnea or tachypnea (12/152, 8%), history of syncope (4/152, 3%), history of pleural effusion (5/152, 3%), exercise intolerance (3/152, 2%), pericardial effusion (1/152, 1%), ascites (1/152, 1%), or other signs (5/152, 3%). Some dogs had more than one reason for evaluation reported. Clinical signs were reported by the owner at the time of evaluation in 36/152 dogs (24%) and included lethargy (15/152), dyspnea or tachypnea (10/152), cough (8/152), exercise intolerance (9/152), syncope (4/152), or other (15/152).

### 3.2. Physical exam

In dogs <1 year of age (*n* = 24), the median body weight was 7 kg (range: 5.5–25.5 kg). In adult dogs, the median body weight was 35.8 kg (range: 25.3–61.1 kg) with males being larger in size (median: 40.2 kg, range: 26.5–61.1 kg) compared to females (median: 32.6 kg, range: 25.3–55 kg). Weight was not recorded in 45 dogs, all but five of which were cardiac screening appointments. A heart murmur was detected in 32% of dogs (49/152), with two dogs reported to have two separate murmurs (the loudest being used for categorization). The murmur was reported as grade I or II in 28/49, as grade III in 10/49, grade IV in 5/49 and grade V in 5/49. Murmur grade was not reported in one dog. The point of maximal intensity was described as left basilar in 20/49, left mid-heart in 1/49, left apical in 17/49, right basilar in 1/49, and right apical in 8/49. The point of maximal intensity was not reported in two dogs. The murmur timing was described as systolic in 43/49, continuous in 4/49 and not reported in two dogs. In 13/49 dogs with murmurs, no underlying structural cardiac disease was identified. Of these 13 dogs, all murmurs were systolic, with the majority graded as soft (grade I: 2/13, grade II: 10/13, and grade III: 1/13), and the most common point of maximal intensity being left basilar (left basilar: 10/13, left apical 1/13, and right apical 2/13). A gallop sound was reported in five dogs (3%). These five dogs were diagnosed with patent ductus arteriosus, DCM, stage B1 MMVD, pulmonic stenosis and one dog was structurally normal. The reason for a gallop sound in the stage B1 MMVD dog and the structurally normal dog is unclear. An auscultable arrhythmia was noted on physical examination in 17 dogs (11%).

### 3.3. Diagnostic testing

Twenty-one of 152 dogs (14%) did not have an echocardiogram performed, with 15 of these dogs being auscultation only cardiac screening appointments in which a heart murmur was not identified and the other six dogs having various combinations of other cardiac tests performed by the respective cardiology service. One of these six dogs had only an ECG and was diagnosed with sinus tachycardia when presenting for evaluation of a distal tibial osteosarcoma. Another dog had an ECG and BP after presenting as a second opinion for systemic hypertension. Three dogs had an ECG and thoracic radiographs (a pre-operative consult for one dog with pleural effusion and right middle lung lobe torsion, a pre-operative consult for one dog with intervertebral disc herniation, and a consult on one dog with suspected aspiration pneumonia). Finally, one dog had an ECG, thoracic radiographs and a BP measurement as part of a consult for acute onset tetraparesis, tachycardia and ventricular arrhythmias. This dog was diagnosed with a hemoabdomen that was confirmed to relate to a liver tumor on subsequent necropsy.

Echocardiograms were performed in the remaining 131/152 dogs (85%). Across the entire study sample, diagnostic ECGs were obtained in 40/152 dogs (26%), thoracic radiographs were obtained in 33/152 (22%), BP measurements were obtained in 29/152 (19%) and Holter monitor data was available in 18/152 dogs (12%).

### 3.4. Echocardiography

Descriptive statistics associated with each echocardiographic diagnoses are presented in Table 2. Of the 131 dogs who had an echocardiogram performed, 65% were structurally normal (85/131). Of Borzoi deemed structurally normal, 40/85 (47%) had trace or mild regurgitation of one or both atrioventricular valves. There were 15 dogs with trace or mild regurgitation of both the mitral and tricuspid valves, while 15 dogs had trace or mild tricuspid regurgitation (TR) only and ten dogs had trace or mild MR only.

**Table 2:** Demographic and arrhythmia frequency data in 131 Borzoi, separated by echocardiographic diagnosis.

**Echocardiographic diagnosis**	**Number of dogs (%)**	**Age (years)**	**Sex (M/F/U)**	***N* (%) of dogs with ventricular arrhythmias**	***N* (%) of dogs with supraventricular arrhythmias**
Structurally normal	85/131 (65%)	4.2 (0.2–11.6)	38/40/7	14/85 (16%)	8/85 (9%)
DCM total	13/131 (10%)	5.7 (0.4–9.7)	9/4/0	6/13 (46%)	4/13 (31%)
DCM: equivocal	4/131 (3%)	3.4 (1–5.7)	2/2/0	2/4 (50%)	0/4 (0%)
DCM: occult	7/131 (5%)	7 (5–8)	5/2/0	3/7 (43%)	2/7 (29%)
DCM: overt	2/131 (2%)	5 (0.4–9.7)	2/0/0	1/2 (50%)	2/2 (100%)
MMVD total	12/131 (9 %)	8.2 (4–10.9)	3/9/0	3/12 (25%)	2/12 (17%)
MMVD: stage B1	11/131 (8%)	8.2 (4–10.9)	3/8/0	3/11 (27%)	2/11 (18%)
MMVD: stage B2	0/131 (0%)	N/A	N/A	N/A	N/A
MMVD: stage C	1/131 (1%)	8	0/1/0	0/1 (0%)	0/1 (0%)
Congenital disease total	19/131 (15%)	2 (0.2–6)	8/10/1	2/19 (11%)	0 (0%)
TVD	4/131 (3%)	2 (1–3)	4/0/0	1/4 (25%)	0/4 (0%)
Patent ductus arteriosus	4/131 (3%)	0.5 (0.2–2)	1/3/0	0/4 (0%)	0/4 (0%)
MVD	3/131 (2%)	1 (1–4.6)	1/2/0	1/3 (33%)	0/3 (0%)
TVD and MVD	2/131 (2%)	1	0/1/1	0/2 (0%)	0/2 (0%)
Equivocal TVD	5/131 (4%)	4 (1.2–6)	2/3/0	0/5 (0%)	0/5 (0%)
Pulmonic stenosis	1/131 (1%)	6	0/1/0	0/1 (0%)	0/1 0%)
Cardiac neoplasia	3/131 (2%)	8.8 (7–9.6)	3/0/0	0/3 (0%)	0/3 (0%)

Age is presented as median (range). DCM, dilated cardiomyopathy; F, female; M, male; MMVD, myxomatous mitral valve disease; MVD, mitral valve dysplasia; N/A, not applicable; TVD, tricuspid valve dysplasia; U, unknown sex.

With regard to acquired cardiac disease, both DCM and MMVD were identified. Dilated cardiomyopathy was diagnosed in 13/131 (10%) Borzoi who had an echocardiogram (equivocal DCM: 4/131, occult DCM: 7/131, overt DCM: 2/131), while 12/131 (9%) were diagnosed with MMVD (stage B1: 11/131, stage B2: 0/131, stage C: 1/131). Nine of 13 (69%) Borzoi affected by DCM were male, while 9/12 (75%) of Borzoi affected by MMVD were female. Eight of 11 Borzoi (73%) diagnosed with DCM that had echocardiographic measurements available for review did not have concurrent end-diastolic dilation of the left ventricle. Select echocardiographic measurements for Borzoi diagnosed with DCM are presented in Table 3 alongside the equivalent data for Borzoi deemed structurally normal. In 11/12 Borzoi diagnosed with MMVD, the mitral valve leaflets were noted to be thickened in the echocardiogram report, with mild prolapse also noted in two of these dogs. No comments about mitral valve leaflet anatomy were recorded in one dog. Of Borzoi with B1 MMVD, three had trace MR, five had mild MR, two had moderate MR and one had severe MR.

**Table 3:** Select echocardiographic measurements from structurally normal Borzoi and Borzoi diagnosed with equivocal, occult, and overt dilated cardiomyopathy.

	**LVIDd (cm)**	**LVIDdN**	**LVIDs (cm)**	**LVIDsN**	**FS%**	**EF%**	**LA:Ao**
Normal (*N* = 85)	4.4 (4.1–4.6) (*N* = 71)	1.54 (1.41–1.62) (*N* = 71)	3.0 (2.7–3.3) (*N* = 71)	0.98 (0.90–1.04) (*N* = 71)	30.1 (27.6–35.2) (*N* = 71)	63.2 (54.9–67.6) (*N* = 23)	1.2 (1.1–1.3) (*N* = 58)
Equivocal DCM (*N* = 4)	4.3 (4.2–4.9) (*N* = 3)	1.54 (1.53–1.74) (N = 3)	3.6 (3.5–3.8) (*N* = 3)	1.19 (1.18–1.24) (N = 3)	17.8 (17.8–20.9) (*N* = 3)	55.4 (*N* = 1)	1.2 (*N* = 2)
Occult DCM (*N* = 7)	5.0 (4.8–5.1) (*N* = 6)	1.72 (1.71–1.74) (*N* = 6)	4.1 (4.0–4.2) (*N* = 6)	1.32 (1.30–1.37) (*N* = 6)	17.3 (14.6–19.8) (*N* = 6)	37.0 (32.0–40.9) (*N* = 4)	1.3 (1.3–1.6) (*N* = 6)
Overt DCM (*N* = 2)^*^	Dog 1: 6.9	Dog 1: 2.23	Dog 1: 6.0	Dog 1: 1.81	Dog 1: 12.3	Dog 1: 33	Dog 1: 2.1
	Dog 2: 4.1	Dog 2: 1.58	Dog 2: 3.5	Dog 2: 1.26	Dog 2: 14.2	Dog 2: 37.2	Dog 2: 2.3

Data are presented as median (interquartile range). The number of available measurements for each category is noted below the associated data.

* Dog 1 with overt DCM was a 9.7-year-old male adult Borzoi and Dog 2 with overt DCM was a 5-month-old male Borzoi puppy. Necropsy of Dog 2 was consistent with DCM.

DCM, dilated cardiomyopathy; EF%, ejection fraction by Simpson's method of discs; FS%, fractional shortening; LA:Ao, left atrial to aortic root ratio; LVIDd, left ventricular internal diameter at end-diastole; LVIDdN, Left ventricular internal diameter at end-diastole normalized to body weight; LVIDs, left ventricular internal diameter at end-systole; LVIDsN, left ventricular internal diameter at end-systole normalized to body weight.

Congenital cardiac disease was diagnosed in 19/131 (15%) dogs. Diagnoses included: tricuspid valve dysplasia (TVD) (*n* = 4), patent ductus arteriosus (*n* = 4), mitral valve dysplasia (*n* = 3; all deemed mild with either mild or trace MR and two having abnormal chordal attachments noted to traverse from the papillary muscles across to the interventricular septum), combined tricuspid and mitral valve dysplasia (*n* = 2; one described as having mild MR and TR with no other valve anatomy comments and one described as having moderate MR and TR with septal tricuspid leaflet tethering), and valvular pulmonic stenosis (*n* = 1, deemed moderate to severe with a transpulmonic velocity of 4.46 m/s). Five additional dogs were deemed equivocal for TVD. Of the five dogs diagnosed with equivocal TVD, four had mild TR, three of which had mild tricuspid valve thickening noted and one of which had equivocal tethering of the septal leaflet noted, and one had mild to moderate TR with no anatomic abnormalities of the tricuspid valve recorded. Comparatively, of the four dogs diagnosed with TVD, one reportedly had mild TR with tethering of the septal tricuspid leaflet, one had moderate TR with tethering of the septal tricuspid leaflet, and thickening, elongation and shortened chordal attachments of the other visible leaflet, one had moderate TR with mild thickening of the tricuspid leaflets, septal leaflet tethering and apical displacement of the tricuspid valve apparatus, and the final dog had mild to moderate TR with moderately thickened leaflets.

Cardiac neoplasia was diagnosed in 3/131 (2%), with two dogs having a mass associated with the right atrium identified and one dog having a heart base mass identified. The dog with a heart base mass also had occult DCM.

### 3.5. Electrocardiography and Holter recordings

Only 48/152 (32%) Borzoi evaluated were screened for arrhythmias with a diagnostic ECG and/or a Holter monitor. Forty dogs had a diagnostic ECG performed, with just over half (21/40) diagnosed with a sinus rhythm, sinus arrhythmia or sinus tachycardia, with no evidence of ectopy or AV block. Ventricular arrhythmias were seen in 14/40 (35%) dogs who had an ECG, as evidenced by single ventricular premature complexes (VPC) in six dogs, complex ventricular arrhythmias (multiform VPC, VPC couplets, triplets or runs of ventricular tachycardia) in six dogs and accelerated idioventricular rhythm in two dogs. Supraventricular arrhythmias were seen in 7/40 (18%) dogs. No dogs had only single supraventricular premature complexes. Supraventricular tachycardia was seen in five dogs and atrial fibrillation was diagnosed in two dogs, both of which had concurrent ventricular arrhythmias and were diagnosed with DCM. A small number of dogs were diagnosed with other arrhythmias (first degree AV block *n* = 1, sinus bradycardia conducting with first degree AV block and periods of accelerated idioventricular rhythm: *n* = 1).

Eighteen dogs had ambulatory ECG monitoring performed with a Holter monitor, with tabular data available for 17. Two dogs had a normal Holter study with an underlying sinus rhythm and no ectopy. Ventricular arrhythmias were seen in 15/18 (83%) Holter studies, as evidenced by single VPCs in seven dogs and complex ventricular arrhythmias (multiform VPC, VPC couplets, triplets or runs of ventricular tachycardia) in an additional eight dogs. Supraventricular arrhythmias were seen in 5/18 (28%) dogs. Two dogs had only single supraventricular premature complexes. Supraventricular tachycardia was seen in 3/18 (17%). Atrial fibrillation was not documented in any dog with Holter monitor data available.

The percentage of ventricular and supraventricular arrhythmias identified during the entirety of the available cardiac evaluation including diagnostic ECG, contemporaneous ECG monitoring during the echocardiogram, and/or Holter monitor data is presented in Table 2. In total, arrhythmias were identified in 34/152 (22%) Borzoi. Eighteen of 85 (21%) structurally normal Borzoi had arrhythmias, including ten Borzoi with ventricular arrhythmias only, four Borzoi with supraventricular arrhythmias only, and four Borzoi with both supraventricular and ventricular arrhythmias. First degree atrioventricular block was noted in two structurally normal dogs that had single ventricular premature complexes, one of which had occasional second degree atrioventricular block as well. Arrhythmias were identified in 8/13 (62%) Borzoi with DCM, with four of these dogs having only ventricular arrhythmias, two having only supraventricular arrhythmias, and two having both ventricular and supraventricular arrhythmias (atrial fibrillation and single VPCs in one occult DCM dog and atrial fibrillation and complex ventricular arrhythmias in one overt DCM dog).

### 3.6. Non-invasive blood pressure measurement

Twenty-nine dogs had a BP measurement recorded in the medical record. Of these 29 dogs, 17 were structurally normal (with or without concurrent arrhythmias), four had stage B1 MMVD, three had congenital heart disease (patent ductus arteriosus, pulmonic stenosis and mild mitral valve dysplasia), three had DCM (one overt and two equivocal) and two did not have echocardiograms. The method of BP measurement was oscillometric in 3/29 dogs and Doppler in the remaining 26/29 dogs. The median systolic BP of all dogs in which a BP measurement was available was 150 mmHg (range: 85–220 mmHg). There were 11/29 dogs (38%) in which the systolic BP measurement was 160 mmHg or greater. If only structurally normal dogs and dogs with stage B1 MMVD were considered (*n* = 21), median systolic BP remained 150 mmHg (range: 85–185).

### 3.7. Thoracic radiography

Of the 33 dogs who had thoracic radiographs performed, eleven were diagnosed as structurally normal (with or without arrhythmias) on echocardiogram, with vertebral heart size (VHS) reported for five dogs as 9.3, 9.5, 9.5, 9.9, and 10.2. Three dogs diagnosed with occult DCM had a VHS reported (9.7, 10.5, and 10.5). Two dogs diagnosed with PDA had a VHS reported (12.0 and 12.2). Lastly, three dogs diagnosed with stage B1 MMVD (one with concurrent ventricular and supraventricular arrhythmias) had a VHS reported (9.6, 10.0, and 10.3).

### 3.8. Follow-up and long-term outcome

Twenty-five out of 152 dogs (16%) had recheck cardiology evaluations. Of these, five dogs had subsequent diagnoses that were not present at initial evaluation. In one 6-year-old male who had a structurally normal heart at the time of initial evaluation (performed after a Holter examination revealed ventricular arrhythmias), occult DCM was diagnosed at subsequent rechecks and treated over the course of 2 years, with ultimate outcome unknown. In another 1-year-old male dog that was structurally normal at the time of a breed screen evaluation, ventricular arrhythmias were diagnosed at age seven. Another 1-year-old male that was diagnosed with TVD at age one was found to have increased estimated pulmonary artery pressure at age four. A 7-year-old male that was structurally normal with ventricular arrhythmias at the time of initial evaluation was found to have stage B1 MMVD at a recheck 1 year later. Lastly, a 2-year-old male diagnosed with a PDA at the time of initial evaluation that underwent surgical ligation of the PDA was found to have developed atrial fibrillation with a normal ventricular response rate at a recheck 2 months after the ligation.

Of the four dogs diagnosed with equivocal DCM, one female with ventricular tachycardia and equivocal systolic dysfunction diagnosed at age one continued to have unchanged systolic function indices and left ventricular dimensions 3.5 years after the original diagnoses, with arrhythmias well-controlled on sotalol. Another male diagnosed with ventricular arrhythmias and systolic dysfunction with normal left ventricular dimensions at age three, continued to maintain similar echocardiographic measurements 4.8 years later, while on chronic therapy with pimobendan and sotalol. Another 5-year-old female with equivocal DCM died at age nine of non-cardiac causes, however she produced a litter of nine puppies over a year after her cardiac evaluation. Three puppies from that litter were reported to have been definitively diagnosed with DCM and one experienced sudden death. The fourth equivocal DCM dog was lost to follow-up.

Of the five dogs that presented for evaluation with a family history of sudden death, all five dogs were diagnosed with ventricular arrhythmias and two had supraventricular arrhythmias diagnosed as well. Four of these five dogs were structurally normal, and one had mitral valve dysplasia with trace MR. One of these dogs died at age 13 of non-cardiac causes and the other four had no follow-up.

The long-term outcome for most cases was unknown. Of 152 dogs in the study, 33 were known to be dead, 22 were known to be living, and 97 had unknown status at the time of writing. Of dogs known to have died, six were considered cardiac deaths, five were unknown, and 22 were non-cardiac. Three of the cardiac deaths were sudden death events and the other three were euthanized (two for refractory CHF and malignant arrhythmias associated with DCM and one for a right atrial mass presumed to be hemangiosarcoma).

One of the sudden death dogs was a 7-year-old male classified as occult DCM at the time of initial evaluation with a concurrent supraventricular tachycardia. A recheck evaluation after starting treatment with diltiazem revealed normalized structure and function after adequate heart rate control, suggesting the systolic dysfunction initially appreciated was related to the tachyarrhythmia. Less than 3 weeks after the recheck examination the dog cried out, collapsed and died. Necropsy revealed multifocal hyaline arteriosclerosis with moderate to severe acute myocardial degeneration, myofibrillar hypertrophy and splitting and moderate epicardial hemorrhage as well as chronic renal lesions and an incidental prostatic carcinoma with no metastases. The attending pathologist deemed that a definitive cause of sudden death could not be ascertained, but the multifocal areas of myocardial degeneration could be the chief contributory factor, noting that the myocardial fibers were predominately affected adjacent to the sclerotic narrowed arteries (of which many were noted), suggesting a compromise in blood supply to the myocardial fibers. The second sudden death dog was normal at the time of cardiology evaluation when he presented at age two for breed-screening. The sudden death event was 16 months after the breed-screen evaluation. The dog was found to have died in his kennel and had been seen normal 3 h prior. A tarp covering his kennel had been accidentally pulled away at some point during the 3-h period, and although the dog had access to water, some degree of heat exposure playing a role in the death cannot entirely be eliminated. Additional family history for this dog revealed that sudden death occurred in a female offspring, great uncle, and direct offspring of the great uncle of this dog. A necropsy was not performed. The final sudden death dog was a 3-year-old male diagnosed with TVD when presenting for evaluation of a heart murmur. The dog had moderate TR with mild thickening of the tricuspid leaflets, septal leaflet tethering and apical displacement of the tricuspid valve apparatus. This dog remained asymptomatic and was reportedly healthy throughout its life but died suddenly at 6.9 years of age. The dog was normal when let outside and was found dead in the yard 5 min later. No necropsy was performed. The owner reported a family history of TVD in the lineage of this dog as well as a half-brother known to have died suddenly at an old age.

Of the three dogs that were euthanized due to cardiac disease, one had a necropsy performed. This dog, a 5-month-old intact male, was euthanized the day of emergency evaluation for DCM, biventricular heart failure, and supraventricular tachycardia. The necropsy reported histologic findings consistent with DCM including subacute, multifocal cardiomyopathy with wavy fibers, cardiomyocyte disarray, fibrofatty degeneration, and myocyte anisocytosis. Histopathological evaluation of the cardiac conduction system was unremarkable.

## 4. Discussion

Borzoi are an uncommon breed presenting to referral veterinary hospitals in the United States, representing a very small percentage of overall canine hospital admissions. As such, it becomes challenging for individual veterinary clinicians to gain a breadth of experience with the breed or appreciate frequency of individual disease diagnoses. These challenges prompted the present multicenter study, as an initial attempt to better define the types and frequency of cardiac disease diagnosed within the Borzoi breed. Of those Borzoi that presented to the referral veterinary hospitals of this multicenter study, 24% were evaluated by a cardiologist, with cardiac breed screening being the reason for evaluation in just over half of the study sample. For cardiologists to optimize cardiac breed screening, knowledge about disease predispositions and spectrum of normal for a given breed is important.

Trace or mild valve insufficiencies of one or both of the atrioventricular valves were reported in nearly half of Borzoi that were deemed structurally normal, with this group having a median age of 4.2 years. As technology associated with echocardiographic machines has improved over time, the prevalence of atrioventricular valve regurgitation identified in healthy individuals has increased, with one human study documenting >80% of people across all age groups as having TR and one-third, one-half and two-thirds of people between 10–19, 20–29, and >30 years of age having MR, respectively (25). Furthermore, human athletes have been reported to have a higher prevalence of TR than non-athletic controls (58 vs. 36%, respectively), potentially related to a small but significant enlargement of the right heart secondary to increased volume load in athletes (26). In another study of elite human athletes, 89.4% had mild TR (27). Borzoi are athletic dogs that compete in a variety of canine athletic competitions including lure coursing, straight racing, and other events. Whether their athletic nature has any bearing on the frequent atrioventricular valve insufficiencies appreciated in this study sample would require further prospective investigation. Breed-specific differences in atrioventricular valve insufficiency frequency may also exist. For example, 45% of healthy Leonberger dogs were found to have trace or mild MR in one recent study (28).

When atrioventricular valve insufficiencies are identified, however, congenital valvular dysplasia or degenerative change must also be considered, particularly in younger or older dogs, respectively. Both TVD and mitral valve dysplasia were reported in these Borzoi, as was MMVD. In fact, TVD was the most common congenital heart disease appreciated in this study, being diagnosed in six dogs (two of whom were diagnosed with concurrent mitral valve dysplasia), with an additional five dogs considered to have an equivocal TVD diagnosis (29). Given the retrospective nature of this study, review of images for standardized assessment and categorization of dysplasia vs. degeneration vs. normal variant was not possible, with dogs categorized based on the original characterization made by the attending cardiologist. In some instances in which dysplasia or equivocal dysplasia were reported in association with trace or mild atrioventricular valve insufficiencies, no valve anatomy comments were recorded. It is possible that some dogs may have been deemed abnormal based on the identification of valve insufficiency alone. On the other hand, it cannot be ruled out that some dogs deemed structurally normal may have had mild anatomic abnormalities that were not appreciated. Given the frequency with which trace or mild atrioventricular valve regurgitation was identified in Borzoi deemed structurally normal by the attending cardiologist, however, classification of tricuspid or mitral valve dysplasia in the absence of any overt valve anatomy abnormalities during breed screen evaluations should be considered cautiously, as these small valve insufficiencies may well be a variant of normal.

With regard to those Borzoi diagnosed with MMVD, dogs tended to be older, and females represented 75% of the affected study sample. The sex distribution in Borzoi is unusual in comparison with the general canine population, where MMVD is 1.5 times more common in males than females (24). Although MMVD was the most common acquired cardiac disease to be diagnosed (*n* = 12), 92% of the Borzoi with MMVD had an early, clinically insignificant stage of the disease (stage B1), with only a single dog documented to have progressed to the point of CHF and no dogs in stage B2. This suggests that MMVD is unlikely to be a frequent clinical concern in this breed.

Dilated cardiomyopathy was also diagnosed in this study sample, and if equivocal cases were combined with occult and overt forms, more Borzoi were found to have DCM (*n* = 13) than MMVD. A diagnosis of DCM is not always straightforward, however. Not only are there multiple causes of a DCM phenotype other than the genetically mediated form of the disease such as diet, toxins, inflammation and tachyarrhythmias, there can also be difficulties in defining the exact echocardiographic criteria required to definitively diagnose the disease. This may be particularly true for sighthounds, where normal echocardiographic reference ranges tend to vary compared to non-sighthound breeds (4, 5). To date, no breed-specific echocardiographic normal reference ranges exist for the Borzoi breed. One study that included a small number of Borzoi (*n* = 9) noted a tendency for left ventricular end-systolic dimensions to exceed non-breed specific 95% prediction intervals (4). If this tendency proves true, it may be particularly relevant, given the importance of left-ventricular end-systolic dimensions in the diagnosis of DCM. For the purposes of this study, one set of echocardiographic criteria was applied to all dogs in which echocardiographic measurements were available for review to attempt to improve consistency in the DCM diagnosis. While newer canine allometric scaling data continues to tighten reference ranges for normalized left ventricular end-diastolic and end-systolic diameters (4, 30), in the absence of breed-specific data, we opted to utilize the wider reference ranges accounted for in earlier canine allometric scaling data (22) to avoid over-diagnosing DCM in a breed that may trend larger, particularly with regard to left ventricular end-systolic dimensions. Prediction intervals for left ventricular end-diastolic and end-systolic volumes in sighthounds have been published (5) but were not utilized to define a DCM diagnosis in this study due to the lower number of dogs in which ejection fraction and left ventricular volume measurements were available in this retrospective dataset. Additionally, due to the retrospective nature of this study, we cannot assume dogs diagnosed in this study sample had a genetically mediated DCM, as detailed history about diet was not consistently available, nor were cardiac troponin levels measured or infectious disease testing pursued in all dogs. In fact, one dog classified as occult DCM appeared to have been a tachycardia-induced cardiomyopathy upon subsequent recheck. Moreover, one dog diagnosed as occult DCM and one diagnosed as equivocal DCM did not have echocardiographic measurements available in the medical record for review, thus they remained as originally categorized by the attending cardiologist. Regardless of these limitations, the data presented here are sufficient to suggest that DCM is a concern in the Borzoi breed, and further prospective research is warranted to better define the prevalence.

Another interesting finding was the prevalence of ventricular arrhythmias, particularly within some of the subgroups of the current study sample. While ventricular arrhythmias are common in dogs with DCM and were appreciated in nearly half of Borzoi diagnosed with DCM in this study sample, they are generally unexpected in dogs with early stages of MMVD. Despite this, 25% of Borzoi with MMVD were found to have ventricular arrhythmias, all of whom had an early form of the disease with no heart enlargement (stage B1). The two dogs with congenital heart disease that had ventricular arrhythmias were considered to have mild TVD and mild mitral valve dysplasia with mild TR and trace MR, respectively. Furthermore, 16% of Borzoi deemed structurally normal were found to have ventricular arrhythmias. This is particularly interesting given that only a minority of the study sample was fully screened for arrhythmias, with only 26% of Borzoi having a diagnostic ECG and only 12% having a Holter monitor performed. The significance of ventricular arrhythmias in these unexpected groups remains unknown, with the potential for either cardiac or non-cardiac disease to be playing a role. In breeds like the Doberman Pinscher, ventricular arrhythmias are a well-documented precursor to DCM and can be used to diagnose the disease (31). Whether the same is true for Borzoi remains unknown, though one structurally normal Borzoi with ventricular arrhythmias in this study was noted to develop occult DCM at a future recheck. Prospective study will be needed to better define the prevalence of ventricular arrhythmias within this breed and to explore the potential relationship with development of DCM.

Lastly, given the concern for sudden death within the Borzoi breed, documentation of both DCM and ventricular arrhythmias within the present study sample represent important areas of interest for future study. Dilated cardiomyopathy and arrhythmogenic right ventricular cardiomyopathy are considered the leading causes of sudden cardiac death in dogs (32), however heritable ventricular arrhythmias leading to sudden death without an obvious underlying structural cause have also been described in a small number of individual dog breeds (33–36). Both of these possibilities should be explored further in the Borzoi breed. Notably, all five dogs who presented for evaluation with a disclosed family history of sudden death were diagnosed with ventricular arrhythmias.

Limitations of the present study are largely related to the retrospective nature of the work. Not all dogs had all cardiac diagnostic tests performed, some dogs had incomplete information available for analysis, and long-term follow up was unavailable for many dogs. Furthermore, standardization of echocardiographic assessments such as atrioventricular valve regurgitation severity was not possible across all centers, with the characterization made by the attending cardiologist. In some instances, trends were noted but limited data precluded definitive conclusions. For example, systolic BP appeared to trend high, similar to what has previously been reported in greyhounds (37–39), however data points were limited. Necropsy data was also very limited, with only one dog that experienced sudden death and one dog with DCM submitted to postmortem examination and no broad conclusions about sudden death or DCM in the breed able to be drawn from these two Borzoi alone. A definitive cause of death in the other sudden death dogs remains unknown. Most of the hospitals who participated in this study were academic veterinary teaching hospitals, thus the study sample may not be reflective of the Borzoi population seen by cardiologists in private practice or by general veterinary practitioners. Additionally, all of the dogs in this study were evaluated in the United States, with results not necessarily applicable to Borzoi from other countries.

In conclusion, although Borzoi are an uncommon breed presenting for evaluation, about one quarter of those that present to referral veterinary hospitals see cardiologists, often for cardiac breed screening evaluations. While both DCM and MMVD were appreciated at similar frequencies, MMVD was almost exclusively mild, while DCM was more likely to result in clinically important disease requiring cardiac medication. Tricuspid valve dysplasia was the most common congenital heart disease diagnosed, however trace to mild atrioventricular valve regurgitation was appreciated in nearly half of structurally normal Borzoi dogs, suggesting careful scrutiny be applied during breed screen evaluations prior to a diagnosis of atrioventricular valve dysplasia. Lastly, despite minimal screening, ventricular arrhythmias were recognized in 19% of dogs that had an echocardiogram, 76% of which had minimal or no structural cardiac disease identified concurrently. This finding warrants further investigation.

## Data availability statement

The original contributions presented in the study are included in the article/supplementary material, further inquiries can be directed to the corresponding author.

## Ethics statement

Ethical approval and client consent was not required by the lead author's institution for a retrospective study in which no research was conducted on live animals.

## Author contributions

KS, SW, and AS were involved in conception of the idea, design, data collection, data analysis and interpretation, and manuscript preparation. ST, BP, AG, NK, JW, EK, LM, AM, JA, SK, and GM were involved in data collection and manuscript preparation. All authors have read and approved the final manuscript.
